# A brief report on Iranian Journal of Basic Medical Sciences in 2013

**Published:** 2014-03

**Authors:** Bizhan Malaekeh-Nikouei

**Affiliations:** 1 Assistant Editor of Iranian Journal of Basic Medical Sciences; 2 Nanotechnology Research Center, School of Pharmacy, Mashhad University of Medical Sciences, Mashhad, Iran

**Figure 1:**
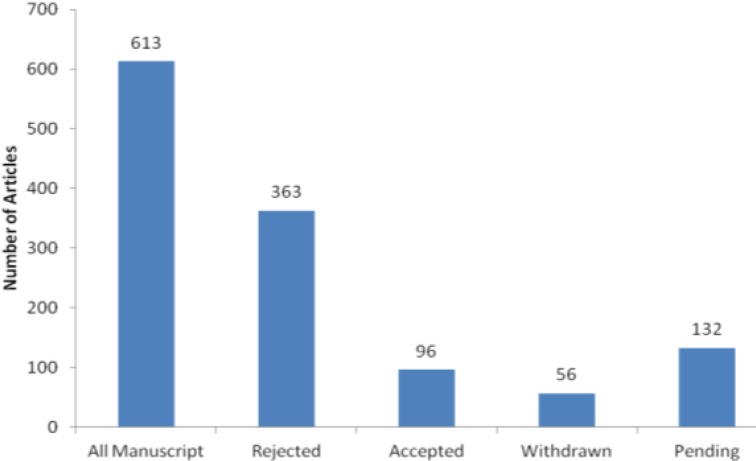
Analysis of manuscripts received in 2013 by Iranian Journal of Basic Medical Sciences

This editorial deals with the state of the Iranian Journal of Basic Medical Sciences (IJBMS) in 2013. In the previous year, we received 613 manuscripts for publication. After peer-review by expert national and international reviewers, 15 % of submitted papers were accepted for publication. As presented in Figure 1, the rejection rate was around 60%. 

**Figure 2:**
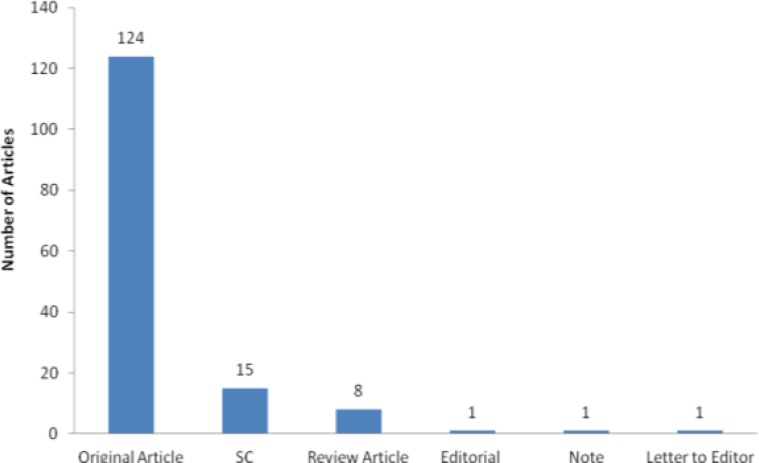
Types of manuscripts published in 2013 by Iranian Journal of Basic Medical Sciences

The publication frequency was doubled by switching from bimonthly to monthly, with around 12 papers per issue. Most of the published papers were original articles; review articles were around 5% of all published papers (Figure 2). 

**Figure 3:**
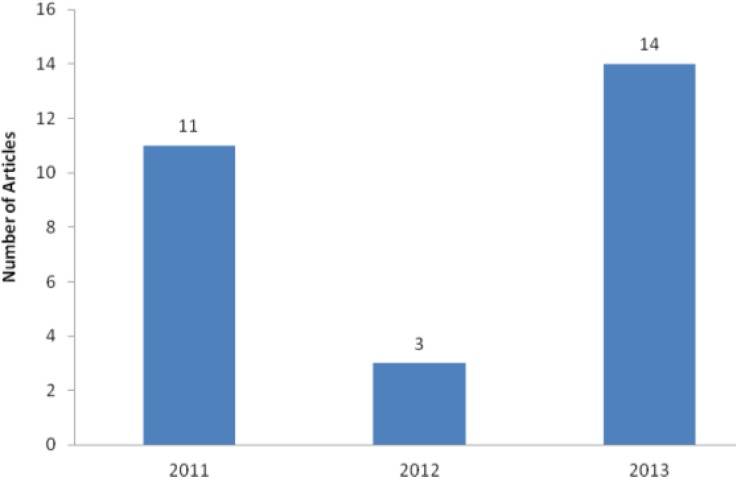
International collaboration in published manuscripts in Iranian Journal of Basic Medical Sciences

Contributions of the international authors were increased compared to the previous years (Figure 3). In 2013, we published papers by corresponding authors from various countries including China, India, Saudi Arabia, Malaysia, Pakistan, Serbia, and Turkey. Contributing authors were from Austria, Canada, Egypt, Lebanon, The Netherlands, Nigeria, and The United Kingdom.

Two special issues were published in 2013. The first one focused on Saffron (*Crocus sativus*). As Saffron is one the most famous plants cultivated in Iran and has various pharmacological effects including anti-asthmatic, anti-carcinogenic, anti-mutagenic, anti-depressant, and immunomodulating and has antioxidant-like properties, it was selected for a special issue. The second issue was conducted with 20 papers on Human T-cell lymphotropic virus-I (HTLV-I), as this virus is endemic in several regions of the Middle East including Iran; various concepts about this virus and related diseases were discussed.

Finally, I should thank all authors and reviewers who contributed to the IJBMS publication process. I hope to publish this journal with better papers and higher quality in 2014. According to the citation analysis done recently, a better impact factor (IF) is expected for the current year.

